# Essential Oil-enhanced digital hypnotherapy for subclinical generalized anxiety: a study protocol for a randomized controlled trial

**DOI:** 10.3389/fdgth.2026.1799921

**Published:** 2026-04-17

**Authors:** Steven Ngandeu Schepanski, Martin Bogdanski, Cornelia Witt, Judith Czakert, Matthias Melzig, Julia Siewert, Wiebke Stritter, Steffen Schulz, Georg Seifert

**Affiliations:** 1 Charité – Universitätsmedizin Berlin, Corporate Member of Freie Universität Berlin and Humboldt-Universität zu Berlin, Charité Competence Center for Traditional and Integrative Medicine (CCCTIM), Berlin, Germany; 2 Charité – Universitätsmedizin Berlin, Corporate Member of Freie Universität Berlin and Humboldt-Universität zu Berlin, Department of Pediatrics, Division of Oncology and Hematology, Berlin, Germany; 3 Freie Universität Berlin, Institute of Pharmacy, Berlin, Germany; 4 Charité – Universitätsmedizin Berlin, Corporate Member of Freie Universität Berlin and Humboldt-Universität zu Berlin, Institute of Social Medicine, Epidemiology and Health Economics, Berlin, Germany

**Keywords:** aromatherapy, autonomic nervous system, digital self-hypnosis, e-mental health intervention, heart rate variability, preventive mental health, relaxation conditioning, subsyndromal anxiety

## Abstract

**Background:**

Subsyndromal generalized anxiety is highly prevalent and associated with impaired well-being, elevated stress, and functional limitations, yet affected individuals often do not meet criteria for guideline-based treatment. Scalable, low-threshold digital interventions that target psychophysiological regulation may help address this gap. Guided self-hypnosis and aromatherapy using essential oils have each demonstrated anxiolytic and relaxation-promoting effects. Combining these approaches may enhance efficacy and allow for conditioning of relaxation responses via olfactory cues.

**Methods:**

This study protocol describes a randomized controlled trial evaluating the efficacy and conditioning potential of a digital self-hypnosis intervention combined with essential oil inhalation in adults with subsyndromal generalized anxiety. A total of *N* = 630 participants will be randomized into six groups. Four groups enter the primary efficacy analysis: (1) self-hypnosis + bergamot essential oil, (2) self-hypnosis + lavender essential oil, (3) self-hypnosis without essential oil, and (4) a minimal-intervention control. The intervention is delivered online over six weeks (Phase 1), followed by a two-week conditioning phase without hypnosis (Phase 2), in which stimulus-specific effects of the essential oils are tested. The primary outcome is subjective relaxation, measured by the Multidimensional Mood Questionnaire (MDBF) at baseline, post-intervention (6 weeks), and post-conditioning (8 weeks). Secondary outcomes include anxiety symptoms, perceived stress, sleep quality, well-being, and worry. In a voluntary subsample, heart rate variability (HRV) and pulse wave variability (PWV) will be assessed as physiological correlates of relaxation. In addition, the questionnaires are expanded to include open-ended questions, enabling an exploratory assessment of participants’ experiences, attitudes, and reflections on the intervention and its potential for sustainability. This approach complements quantitative results with qualitative insights and may reveal new perspectives for future research.

**Discussion:**

This study is expected to provide evidence on the efficacy of essential oil-enhanced digital hypnosis for subsyndromal anxiety and will examine whether repeated pairing of hypnosis and olfactory stimulation induces conditioned relaxation responses. If effective, this multimodal, low-intensity intervention could represent a scalable preventive approach for individuals with increased anxiety who are not receiving formal treatment and have been medically diagnosed.

**Clinical Trial Registration:** https://www.drks.de/search/de/trial/DRKS00039047/details, Identifier DRKS00039047.

## 1 Introduction

Adults with subsyndromal generalized anxiety often experience significant distress and impairment comparable to those with clinical generalized anxiety disorder (GAD) (1). Despite this burden, these individuals may not qualify for standard treatments and thus benefit from accessible, preventive interventions. Self-help techniques like relaxation training, mindfulness, or hypnosis delivered digitally offer promising avenues for supporting this group. In particular, hypnosis and hypnotherapy have demonstrated effectiveness in reducing anxiety and stress. Reviews find that hypnotherapy can significantly lower anxiety levels and simultaneously modulate the autonomic nervous system by reducing sympathetic arousal and increasing parasympathetic tone (2). This physiological shift under hypnosis is associated with relaxation and could potentially be cardioprotective (2).

Aromatherapy with essential oils is another complementary strategy shown to alleviate anxiety. For example, inhalation of lavender (*Lavandula angustifolia Mill.*) essential oil has repeatedly been found to reduce stress and anxiety and improve mood in various populations (3–5). Likewise, bergamot (*Citrus bergamia*) essential oil-rich in limonene, linalool, and linalyl acetate-has demonstrated calming, anxiolytic effects. Clinical studies report that bergamot oil aromatherapy can lower heart rate and blood pressure and reduce stress and anxiety levels (6–8). These essential oils are generally safe and well-tolerated, making them suitable for a wide application in anxiety relief settings (9). Importantly, lavender and bergamot have distinct aromatic profiles and chemical compositions, allowing researchers to compare their effects on relaxation. Both oils' anxiolytic properties, documented in the literature, provide a strong rationale for their use in a stress-reduction intervention.

Combining guided self-hypnosis with essential oil inhalation may yield synergistic benefits for relaxation. Hypnosis can induce a deep relaxation response, and pairing this state with a specific olfactory stimulus (the essential oil's aroma) opens the door for classical conditioning of relaxation. Through repeated pairings, the neutral scent of an essential oil could become a conditioned stimulus (CS) that evokes calmness even in the absence of hypnosis. In fact, prior work has explored hypnotherapeutic olfactory conditioning, where patients undergoing hypnosis learn to associate a pleasant odor with feelings of safety and control (10). Preliminary results using this technique in cases of post-traumatic stress showed promise that the scent alone later helped induce relaxation and reduce anxiety (10). This concept aligns with fundamental Pavlovian conditioning principles and suggests that a well-timed combination of aromatherapy and hypnosis could train individuals to self-soothe by simply inhaling the scent after conditioning.

The present study builds upon a successful trial in healthy individuals (11) and aims to rigorously test a multimodal relaxation intervention in subsyndromal anxiety. The predecessor study was a four-arm randomized trial with over 500 participants, which found that combining web-based self-hypnosis with an essential oil significantly increased subjective relaxation compared to either technique alone. Participants who received the combined hypnosis plus essential oil reported notably greater improvements on relaxation scales with moderate effect sizes relative to controls. Encouraged by these findings, this study will extend this approach to individuals with mild generalized anxiety symptoms and examine not only the acute efficacy but also the conditioning effect of the essential oil over time. In summary, the study's primary goal is to evaluate whether digital self-hypnosis combined with inhalation of an essential oil, either lavender or bergamot, can produce greater relaxation and anxiety reduction than hypnosis alone or minimal intervention, and secondarily to determine if the essential oil's scent can become a stand-alone cue for relaxation after repeated pairing with hypnosis as well as whether objective physiological measures such as heart rate variability (HRV) can prove a relaxation effect on the autonomic nervous system.

## 2 Methods and analysis

### 2.1 Study design

**Figure 1:**
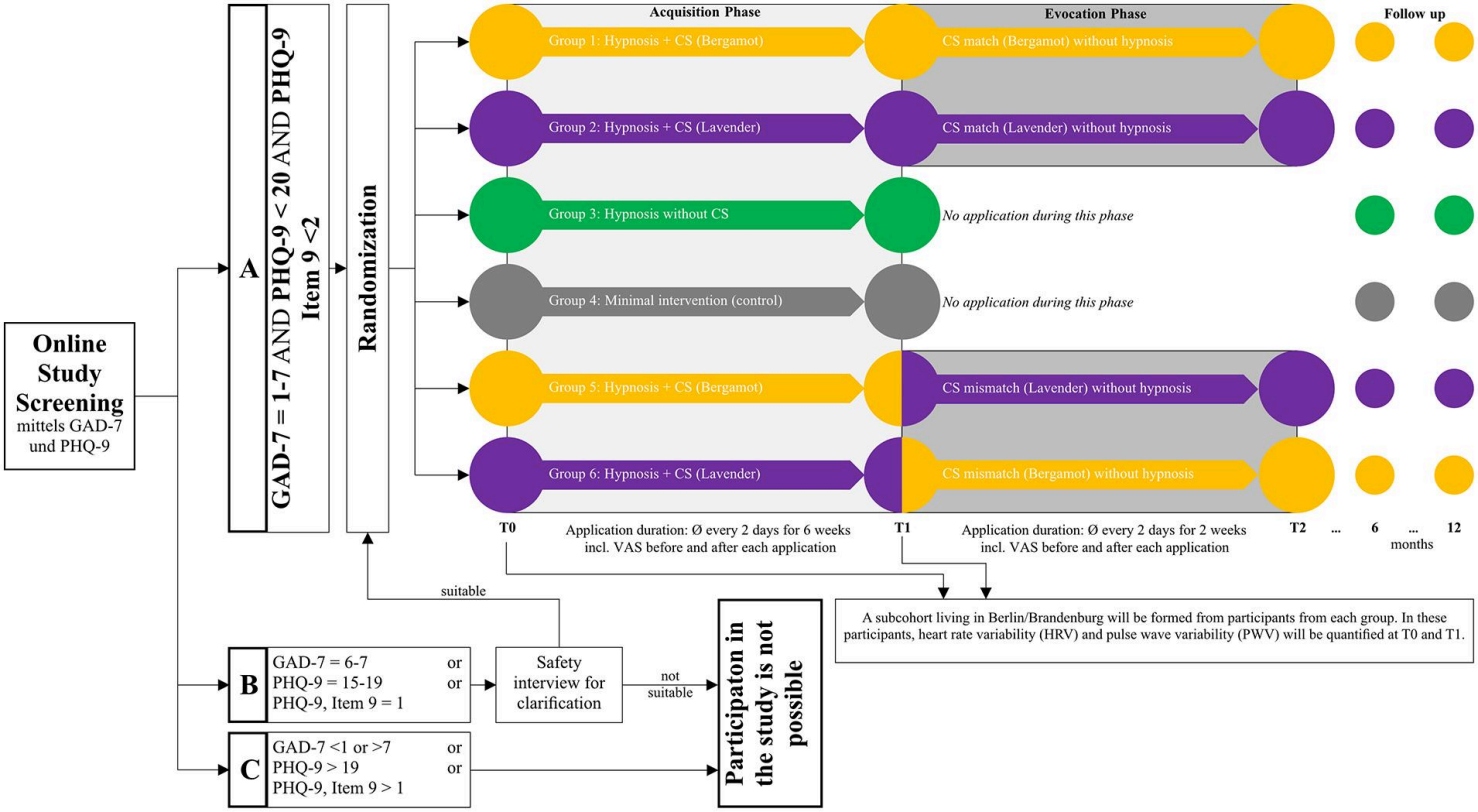
Study overview and timeline. After online screening (GAD-7/PHQ-9), eligible participants are randomized (1:1:1:1:1:1) to six arms. Phase 1 (Acquisition; 6 weeks) involves self-hypnosis every ∼2 days, with or without essential oil exposure depending on group; pre/post-session relaxation is recorded via VAS. Phase 2 (Evocation; 2 weeks) tests conditioned responding: participants in aroma arms complete essential-oil exposure without hypnosis every ∼2 days (CS match = same oil as in Phase 1; CS mismatch = switched oil), with VAS recorded pre/post exposure. Assessments are conducted at baseline (T0), post-phase 1 (T1), and post-phase 2 (T2); follow-up surveys occur at 6 and 12 months without further application. An optional Berlin/Brandenburg subcohort undergoes exploratory physiological assessment (HRV, PWV) at the time points indicated. CS, conditioned stimulus; GAD-7, generalized anxiety disorder-7; PHQ-9, patient health questionnaire-9; HRV, heart rate variability; PWV, pulse wave variability; VAS, visual analogue scale.

This study is designed as a randomized, controlled trial with six parallel groups, conducted fully online, except for optional physiological measurements (Figure 1). The intervention spans 8 weeks in two phases. Phase 1 (weeks 1–6) involves guided self-hypnosis sessions delivered via an online platform (SoSci Survey, Version 3.5.02, SoSci Survey GmbH, Munich, Germany) with concurrent essential oil aromatherapy in certain groups. Phase 2 (weeks 7–8) involves exposure to the essential oil alone without hypnosis to test conditioned relaxation responses. The trial was registered in the German Clinical Trials Register (Deutsches Register Klinischer Studien, DRKS; ID: DRKS00039047) on January 20, 2026. All procedures were approved by the Institutional Review Board of Charité – Universitätsmedizin Berlin (Ethikkommission der Charité, Campus Charité Mitte, reference number EA2/244/25) and were conducted in accordance with the Declaration of Helsinki (1975, as revised in 2008) and relevant local data protection regulations.

### 2.2 Participants

The target population is adults (18–65 years) in Germany with subsyndromal generalized anxiety. Key inclusion criteria are: (a) GAD-7 anxiety questionnaire score between 1 and 7, indicating minimal to mild anxiety, not meeting GAD diagnostic threshold, (b) no acute psychiatric condition requiring treatment, and (c) willingness and ability to follow the 8-week intervention schedule online. Participants must reside in Germany, have sufficient German language skills, since all materials are in German, and provide informed consent online. To ensure safety, individuals are excluded if they screen positive for any serious mental health issues, such as moderate-to-severe anxiety or depression [e.g., GAD-7 ≥15, Patient Health Questionnaire (PHQ)-9 ≥20, or PHQ-9 suicidal ideation item > 1, which suggest clinical severity] (12, 13), current psychotherapy or psychiatric treatment, or a history of conditions like psychosis, bipolar disorder, post-traumatic stress disorder, or severe personality disorder. Participants with known scent sensitivities or olfactory impairments are also excluded specifically for anyone with an intolerance or aversive reaction to lavender or bergamot essential oil, or diagnosed anosmia (loss of smell), cannot take part. This screening is implemented via an initial online questionnaire (including GAD-7 and PHQ-9) before randomization. Only those who pass the screening thresholds and report no disqualifying conditions are granted access to the study portal. This procedure ensures the sample truly comprises people with subclinical anxiety levels and avoids enrolling anyone in need of more intensive treatment. If a prospective participant's responses indicate high symptom severity or risk, the system automatically excludes them and provides standardized feedback recommending appropriate help such as contacting a physician or therapist, thereby prioritizing safety.

### 2.3 Recruitment

Participants will be recruited from the general public in Germany, reflecting the subsyndromal nature of the target condition, which typically does not lead to routine clinical referral. Recruitment will be conducted via online channels (e.g., social media advertisements, study-participation platforms) and offline postings (e.g., flyers in primary care practices, dental practices, pharmacies, and community settings such as libraries, community centers, and health- and mindfulness-related venues). Interested individuals will access an online screening survey (GAD-7, PHQ-9 and eligibility items); only eligible participants will be enrolled and randomized. Participation is voluntary and not remunerated. As non-financial compensation, participants in aromatherapy arms will keep the essential oil stick and essential oil, and all participants will receive access to the self-hypnosis audio files after completing the final assessment, to support continued self-care if desired.

### 2.4 Safety procedures and participant contact

The intervention is fully self-administered via the online platform; no therapeutic interaction is provided. Participants can contact the study team at any time for study-related questions. Eligibility is determined through a rule-based online screening (GAD-7, PHQ-9, treatment status, and safety-relevant history). Individuals exceeding predefined safety thresholds (e.g., GAD-7 outside the inclusion range; PHQ-9 ≥20; PHQ-9 item 9 > 1; or self-reported history of psychosis, bipolar disorder, post-traumatic stress disorder (PTSD), or severe personality disorder) are automatically excluded and receive standardized information recommending appropriate professional support and crisis resources. In rare borderline or ambiguous cases, a brief structured safety call (approx. 3–5 min) is conducted by psychology-trained study staff to confirm that participation in a low-intensity self-help intervention is appropriate. This safety call is not diagnostic or therapeutic. Enrollment proceeds only if no indications of treatment-requiring symptom severity are present.

### 2.5 Interventions

#### 2.5.1 Procedure

In Phase 1 (weeks 1–6), participants practice digital self-hypnosis every two days (approx. 3–4 sessions per week) using audio recordings delivered through the SoSci web interface. Each session involves listening to a guided hypnosis audio; the first session is ∼22 min long, and subsequent sessions last ∼11 min each. The hypnosis script focuses on inducing relaxation and calm, those used in the earlier study (11), and it is standardized across participants aside from the experimental manipulations of essential oil. Participants are instructed to find a quiet, comfortable place for each session and to listen to the audio using headphones or speakers without interruption.

Simultaneously, groups 1, 2, 5, and 6 combine these hypnosis sessions with aromatherapy using an essential oil. Those assigned to an aromatherapy condition receive a small aroma stick (inhaler) infused with either Lavender essential oil or Bergamot essential oil, depending on group, to use during the sessions. They are instructed to inhale from the aroma stick at the beginning of each hypnosis session and intermittently breathe in the fragrance as needed throughout the audio, allowing the olfactory stimulus to be present during the hypnotic relaxation. By pairing the specific essential oil with the hypnosis-induced relaxation repeatedly over 6 weeks, the intervention seeks to create a strong mental association between that smell and the relaxed state.

After six weeks of regular practice, Phase 1 concludes with the T1 assessment. Immediately thereafter, Phase 2 (weeks 7–8) begins, lasting two additional weeks. In Phase 2, participants no longer do the hypnosis sessions; instead, they engage in essential oil exposure alone every two days, continuing with the same schedule of thrice weekly exposure. During these sessions, they will inhale from the aroma stick, containing an essential oil, for a brief period (several minutes) but without any accompanying audio or hypnosis. The purpose is to test whether the essential oil itself can elicit a relaxation response after the conditioning in Phase 1. Some groups will receive the same oil in Phase 2 as they did during Phase 1 (matched condition), while others will receive a different oil (mismatched condition) to test the stimulus specificity of the conditioned response. No explicit relaxation exercise is given in this phase aside from the essential oil exposure. Participants still rate their mood before and after each essential oil exposure, as described below, to gauge any relaxation effect purely from the scent.

#### 2.5.2 Essential oil dosing and essential oil stick standardization

To ensure consistent dosing across participants, aromasticks (PRIMAVERA LIFE GmbH, Germany; Article No. 60009) with standardized absorbent cotton wicks (100% cotton fleece) will be used. These aroma sticks are designed for self-filling and controlled inhalation and are supplied with identical wick material and housing components. Participants will be instructed to apply 3–5 drops of the assigned essential oil to a fresh cotton wick, in accordance with the manufacturer's standardized instructions. The impregnated wick will then be inserted into the aroma stick and sealed using the designated closure mechanism. This dose range corresponds to the manufacturer's recommended dosage for personal inhalation use. Participants will receive standardized instructions for aromastick handling and use illustrated as step-by-step guide included in the shipment.

Based on manufacturer guidance and prior experience, the essential oil intensity of a single application persists for approx. two days, after which participants will be instructed to re-apply the same standardized dose using a new wick. This dosing schedule corresponds directly to the intervention frequency (every two days), ensuring consistent exposure across participants and sessions.

All participants will receive identical aroma stick kits, essential oils, and illustrated step-by-step instructions based on the manufacturer's original usage protocol. Participants will confirm correct preparation and refill procedures via a checklist integrated into the online study platform. The selection of essential oils and dosing procedures was informed by expertise in pharmacognosy and aromatherapy within the research team and supported by the product-specific handling recommendations of the supplier. The supplier had no role in study design, data collection, analysis, interpretation, or publication decisions.

### 2.6 Study groups

Participants are randomly assigned to one of six groups (arms) in a 1:1:1:1:1:1 ratio. Four groups are primary comparison arms for the main efficacy analysis at 6 weeks, and two additional groups are included for secondary analyses of conditioned stimulus specificity in Phase 2.

**Group 1: Hypnosis** + **Essential Oil (Bergamot).** Participants receive the self-hypnosis audio and inhale bergamot essential oil during each session in Phase 1. In Phase 2, they continue with bergamot oil only (no audio) on the same schedule. This is a conditioned stimulus (CS)-match condition where the same essential oil is used for conditioning and testing.

**Group 2: Hypnosis** + **Essential Oil (Lavender).** Participants receive the hypnosis audio plus lavender essential oil inhalation during Phase 1 sessions. In Phase 2, they use lavender oil only without hypnosis. This is the CS-match for lavender.

**Group 3: Hypnosis Only (No Oil).** Participants receive guided hypnosis sessions in Phase 1 with no aromatherapy. This controls for the effects of hypnosis alone. They do not use any aroma in Phase 2 either and effectively have no active intervention in weeks 7–8.

**Group 4: Minimal Intervention Control.** Participants do neither hypnosis nor essential oil in Phase 1. Instead, they may receive a minimal activity just to match time/expectation. This arm serves as a baseline and expectancy control. They also do not receive any aroma in Phase 2.

**Group 5: Hypnosis** + **Oil (Bergamot) → Switch to Lavender.** These participants start identical to Group 1 (hypnosis + bergamot oil in Phase 1). However, in Phase 2 they experience a CS-mismatch by switching to lavender without hypnosis. This group helps test if a different scent can trigger relaxation or if the effect is lost when the conditioned stimulus changes. It serves as a control for Group 1 in the conditioning analysis.

**Group 6: Hypnosis** + **Oil (Lavender) → Switch to Bergamot.** These participants mirror Group 2 initially (hypnosis + lavender in Phase 1), then switch to bergamot oil only in Phase 2, creating a CS-mismatch. This serves as a control for Group 2 to assess stimulus specificity.

All groups 1, 2, 5, and 6 receive the full combined intervention (hypnosis + essential oil) in Phase 1, ensuring that Groups 5 and 6 have the same conditioning opportunity as their counterparts before the switch. Groups 3 and 4 allow us to isolate the effects of hypnosis itself (Group 3) and to account for any changes over time not attributable to the interventions (Group 4). Randomization is implemented via the SoSci Survey platform's built-in algorithm once participants are deemed eligible, ensuring allocation concealment. Because of the nature of the interventions, participants will know if they are using an oil or not (blinding is not feasible on the user level), but they will not be informed of the study hypotheses regarding which combination is expected to be most effective. The trial is single blinded in that the data analysts/statisticians will remain blind to group labels until after the primary analyses are completed, to prevent bias in outcome analysis.

### 2.7 Timeline and assessments

The study duration for each participant is 6 weeks from start to finish and 8 weeks in total for participants from groups 1, 2, 5, and 6, with three main measurement points: baseline (T0), post-Phase 1 (T1 at 6 weeks), and post-Phase 2 (T2 at 8 weeks). Additionally, brief self-reports are collected throughout the intervention period to track immediate effects and adherence.

**Week 0: Baseline (T0).** Upon enrollment and after screening, participants complete an online baseline questionnaire battery before any intervention. This includes demographic information, the Multidimensional Mood State Questionnaire (MDBF) (14), the GAD-7 (13), the Perceived Stress Scale (PSS-14) (15), the Pittsburgh Sleep Quality Index (PSQI) (16), the WHO-5 Well-Being Index (17), and the Penn State Worry Questionnaire (PSWQ, German short form) (18). These instruments establish baseline levels of relaxation, anxiety severity, perceived stress, sleep quality, psychological well-being, and trait worry, respectively. A subset of participants in Berlin also undergoes baseline physiological recording (continuous electrocardiogram (ECG) and pulse). The intervention commences on the day following completion of the baseline assessment.

**Weeks 1–6: Intervention Phase 1.** Participants follow the assigned intervention every two days (∼3 times per week). Before each session, they are prompted to rate their current subjective relaxation level using a simple Visual Analogue Scale (VAS) (e.g., “How relaxed do you feel right now?” on a 0–100 scale). Immediately after each session, whether hypnosis with or without essential oil, they rate their relaxation again on the VAS. These pre-post VAS measures for every session provide a running check of acute changes in relaxation per session and allow analysis of trends. During this phase, Groups 1, 2, 5, 6 use their essential oil aroma sticks during the hypnosis sessions as instructed, while Group 3 uses no essential oil (hypnosis only) and Group 4 simply continues with minimal intervention. The study platform tracks adherence.

**Week 6: Post-Intervention Assessment (T1).** At the end of week 6, after the last Phase 1 session, all participants complete the T1 online survey. This repeats the same set of standardized questionnaires as at baseline (MDBF Calmness-Restlessness, GAD-7, PSS-14, PSQI, WHO-5, PSWQ) to quantify changes in relaxation and secondary outcomes from T0 to T1. In addition, participants are asked an open-ended question about their experience by asking for example, “Please describe how you experienced the intervention.” and “How did you integrate the intervention into your daily life?” This qualitative prompt allows them to reflect on any perceived benefits, challenges, or routine they developed over the six weeks. The responses, entered as free text, will later be analyzed to gain insights into engagement, acceptability, and subjective impact. This approach further allows for the identification of unforeseen themes that may emerge in connection with the intervention. Participants in the physiology subgroup come into the lab around this time for their second physiological measurement.

**Weeks 7–8: Conditioning Phase 2.** These two weeks focus on testing conditioned relaxation. Participants in Groups 1, 2, 5, 6 now use the aroma stick alone every two days, without using the hypnosis audio. Group 1 continues with bergamot essential oil inhalation; Group 2 with lavender oil, both maintaining the same CS as in conditioning (match). Group 5 switches to lavender oil only, after being conditioned with bergamot, and Group 6 switches to bergamot oil only, after conditioning with lavender. Group 3 and Group 4 will not be followed up in this phase. As in Phase 1, participants in the active arms (1, 2, 5, 6) will record a VAS relaxation rating before and after each aroma exposure to quantify any immediate relaxation effect from the scent. This data will show whether merely inhaling the familiar essential oil produces a significant calmness boost, and whether that holds true only for the matching scent or also occurs with a mismatched scent).

**Week 8: Final Assessment (T2).** At the end of week 8, all participants, including groups 3 and 4, complete a T2 survey which again includes the MDBF Calmness-Restlessness subscale and the secondary outcome questionnaires (GAD-7, PSS-14, PSQI, WHO-5, PSWQ). This final measurement captures any further changes after the Phase 2 period.

Additionally, long-term follow-ups are planned at approx. 6- and 12-month post-intervention. Participants will be contacted via email to fill out a brief online follow-up survey. These follow-ups will primarily include open-ended questions asking participants to reflect on any lasting changes or benefits they attribute to the intervention, such as “How has your well-being changed in the months since participating in the study?”, “How has handling of stress changed in the months since participating in the study?” or “Have you continued to use any techniques or experienced any long-term effects?”. The responses will be analyzed qualitatively to explore the sustainability of the intervention's impact. These follow-ups are exploratory and voluntary; data will be pseudonymized and used to gauge any extended outcomes beyond the controlled trial period.

### 2.8 Physiological subgroup

To complement self-reported outcomes with physiological data, the study includes an optional physiology component for a subset of participants. Up to 15 participants per group (approx. n≈90 total) who live in the Berlin/Brandenburg region will be invited to attend laboratory sessions at the Charité – Virchow Clinic Campus in Berlin. Electrophysiological measurements will be performed over a 15-min period during which the participant is at rest and in a supine position in the electrophysiological laboratory. During this period, the ECG and pulse are recorded using wearables. Electrophysiological measurements are to be recorded at two time points: initially at the baseline (T0) and subsequently following the six-week intervention period (T1). The ECG and pulse raw data indices were utilized to derive HRV and pulse wave variability (PWV), which are indices of autonomic nervous system (ANS) activity. The activity of the sympathetic nervous system (SNS), the parasympathetic nervous system (PNS), or both components of the ANS is indicated by these indices. In the course of each and every recording session, the participants were equipped with a Polar H10 chest-strap heart rate sensor (for the purpose of recording ECG for HRV) and a Polar Verity Sense optical pulse sensor (for the purpose of PWV via photoplethysmography). It is hypothesized that the response to the various 6-week interventions would be subject to change, and that the before-and-after effect would vary in the autonomous variables depending on the nature of the intervention. This methodology facilitates the representation of variation in dynamic changes across groups and over time. A comparison of the time points T0 and T1 will facilitate objective and quantitative mapping of the dynamic changes caused by the interventions. The autonomous measurements will facilitate the characterization of the interventions in terms of their mechanisms of action, and the extent to which each intervention leads to the assumed subjective improvement in relaxation/recovery (increased PNS). In addition, the study will elucidate the way this intervention enhances autonomic-vegetative function in the short term. The utilization of HRV and PWV in the time domain, in the frequency domain and nonlinear dynamics, has been demonstrated to be a reliable indicator of changes in sympathovagal balance (19). A greater HRV (particularly high-frequency HRV) and a slower PWV (which reflects vasodilation and reduced arterial stiffness) would suggest a shift towards parasympathetic dominance and relaxation. It is hypothesized that the combination of hypnosis and essential oils may result in increased relaxation, which is further enhanced by the intervention and over time from T0 to T1 in comparison to the hypnosis-only group. This is consistent with the hypothesis that deeper or more effective relaxation training is achieved through this method. The resultant phenomenon is evidenced by a decline in sympathetic tone and an augmentation in vagal tone, culminating in a state of profound relaxation and recuperation through the deactivation of the ANS.

However, given the small subgroup sample, these physiological analyses are exploratory. The measurements are carefully scheduled to avoid acute effects of a session (participants do not do a hypnosis session right before the measurement; it's a resting baseline capture). The present sub-study offers an objective evaluation of the intervention's impact on bodily stress arousal, in addition to its subjective effects.

### 2.9 Outcomes

The primary outcome of the trial is subjective relaxation, operationalized as the score on the “Calmness-Restlessness” subscale of the Multidimensional Mood State Questionnaire (MDBF) (14). This subscale directly assesses the participant's current state of inner calm vs. agitation, making it a suitable primary endpoint for an intervention aiming to increase relaxation. The MDMQ has demonstrated good internal consistency in prior research, with Cronbach's *α* between 0.86 and 0.94., depending on the population and setting (14). Primary analysis focuses on the change in this relaxation score from baseline (T0) to post-intervention (T1, week 6). The key comparison is between the combined Hypnosis + Essential Oil groups (1 and 2) and the control conditions (Hypnosis-only and Minimal Intervention). Several secondary outcomes will also be examined (all measured at T0, T1, and T2 using validated self-report instruments).

**Anxiety symptoms.** GAD-7 (13), a brief scale measuring the frequency of core GAD symptoms (worry, tension, etc.) in the past two weeks. It will indicate if the intervention reduces overall anxiety levels.

**Perceived stress.** PSS-14 (15) gauges the extent to which situations in life are appraised as stressful. We will see if participants report feeling less overwhelmed by stress after the intervention.

**Sleep quality.** PSQI (16) assesses sleep patterns and disturbances over the past month. Given the calming nature of the intervention, improvements in sleep quality (lower PSQI scores) are expected in the active groups, as anxiety and sleep are often linked.

**Well-being.** WHO-5 Well-Being Index (17), a short measure of positive mood and vitality. This captures any boost in general mental well-being or mood that might accompany stress reduction.

**Worry tendency.** PSWQ (20), German short form (PSWQ-d) measures trait worry severity. We will explore whether regular relaxation practice and perhaps improved coping reduce habitual worry levels in participants.

In addition to these standardized measures, intervention adherence and acceptance will be evaluated through a custom questionnaire at T1. This includes items about how often participants actually used the intervention as instructed (adherence), how easy or practical it was to integrate into daily life, any barriers encountered, and subjective perceived benefit or satisfaction with the intervention. These data will be reported descriptively to assess feasibility and user acceptance of this digital self-help approach. There is also an exploratory “worry context” checklist module: participants indicate the domains of life in which they currently experience the most worry such as work, relationships, health, and finances. While not an outcome *per se*, this can help describe the sample and potentially stratify whether the intervention effects differ by worry context.

From the Phase 2 conditioning period, the primary outcomes of interest remain subjective relaxation (MDBF Calmness) and VAS relaxation ratings. The critical comparison here is between matched vs. mismatched scent groups. If classical conditioning is successful and stimulus-specific, Groups 1 and 2, who get the same essential oil in Phase 2 as they had during conditioning, should show a greater relaxation response than Groups 5 and 6, who receive a different scent in Phase 2. In contrast, if any scent simply produces a general context effect, the mismatch groups might also relax, suggesting a more generalization or context effect rather than a specific conditioned link. Group 3 (hypnosis only) and Group 4 (minimal control) provide additional reference points at T2 such as Group 3 might show if any residual benefit of hypnosis persists without further sessions, and Group 4 indicates any natural change over 8 weeks without intervention.

### 2.10 Hypotheses

**Primary Hypothesis (H1—Efficacy).** After six weeks of intervention (T1), participants receiving self-hypnosis combined with essential oil inhalation (Groups 1 and 2) will demonstrate greater improvement in subjective relaxation, as measured by the Multidimensional Mood Questionnaire (MDBF), compared to participants receiving self-hypnosis without essential oil (Group 3) or a minimal-intervention control (Group 4).

**Primary Null Hypothesis (H0).** There are no differences between intervention groups in change in subjective relaxation from baseline to T1. **Primary Alternative Hypothesis (H1).** At least one intervention group differs from the others with respect to change in subjective relaxation from baseline to T1.

**Secondary Hypothesis 2.1 (Conditioned Relaxation).** In Phase 2, the essential oil alone will elicit relaxation in those groups that have consistent pairing in Phase 1. Specifically, participants in the CS-match conditions (Group 1 with bergamot, Group 2 with lavender) will show a significant increase in relaxation as measured by pre-post VAS and possibly MDBF from T1 to T2, when exposed to the essential oil alone, whereas those in CS-mismatch conditions (Group 5 and 6, who receive a different oil than used in training) will have smaller or no relaxation response. We hypothesize a stimulus-specific conditioning effect, meaning the relaxation response is tied to the particular essential oil used during training, not just any pleasant essential oil.

**Secondary Hypothesis 2.2 (Broader Psychometric Benefits).** The combined hypnosis + essential oil intervention will lead to greater improvements in other psychological outcomes compared to hypnosis alone or minimal intervention. We expect to see reductions in self-reported anxiety symptoms (lower GAD-7 scores), perceived stress (lower PSS-14), and worry (lower PSWQ-d) as well as improvements in sleep quality (lower PSQI) and overall well-being (higher WHO-5) in Groups 1 and 2 vs. Groups 3 and 4 at T1. This hypothesis posits that beyond immediate relaxation, the 6-week intervention confers broader mental health benefits (less anxiety and stress, better sleep and mood), echoing findings that combined mind-body interventions can positively affect multiple domains of functioning.

**Exploratory Hypothesis 2.3 (Moderators of Effect).** We will explore whether certain baseline factors moderate the intervention's effectiveness. Demographic factors like age, gender, or socioeconomic status might influence outcomes. For instance, it's possible that participants with higher initial anxiety, though still subthreshold, benefit more or less from the intervention, or that younger vs. older participants have different adherence rates. These analyses are exploratory and will be used to generate hypotheses for which subgroups respond best to this approach.

**Exploratory Hypothesis 2.4 (Physiological Effects).** We hypothesize that the combination of hypnosis and aromatherapy yields measurable physiological relaxation, reflected in increased parasympathetic activity or reduced sympathetic activity compared to hypnosis alone. In the laboratory subgroup, it is hypothesized that Group 1/2 (combined) will demonstrate larger increases in HRV, particularly high-frequency HRV, which is indicative of parasympathetic tone, and/or a greater reduction in heart rate and PWV from T0 to T1 than Group 3 (hypnosis only). This would suggest an additive effect of the essential oil on the body's relaxation response, consistent with objective evidence that an essential oil can modulate autonomic activity. However, given sample size limitations, this will be an exploratory outcome.

**Exploratory Hypothesis 2.5 (VAS Trajectory and Learning Effects).** By examining the VAS relaxation ratings collected before and after each session, we hypothesize that participants receiving the combined intervention will not only have larger acute relaxation gains per session on average than those in comparison groups, but they may also exhibit learning effects over time. A learning effect would manifest as gradually higher pre-session relaxation levels as weeks progress the participant's baseline state before each session improves, indicating they carry over some relaxed state or become more skilled at self-relaxation. We expect such effects might be most pronounced in those who undergo conditioning (Groups 1 and 2), potentially showing an upward drift in pre-session VAS scores and/or a plateau indicating habituation to relaxation. We will explore and graph these trajectories for insight into how the relaxation response develops with practice.

**Exploratory Hypothesis 2.6 (Qualitative Outcomes).** Through qualitative feedback, the open-ended responses at T1 and follow-up, we expect many participants to report positive experiences such as increased awareness of their mental well-being, incorporation of relaxation techniques into daily life, or newfound coping strategies. This approach also allows for the inclusion of potential negative experiences and challenges. We will qualitatively analyze these narratives for common themes. One exploratory question is whether participants experienced continued use of the essential oil or hypnosis techniques beyond the study and any long-term benefits or drawbacks. For example, some may report that the scent of lavender or bergamot now readily helps them calm down during stressful moments, indicating successful conditioning, or that the study motivated them to pay more attention to self-care. These rich personal insights will help assess the real-world impact and sustainability of the intervention beyond numeric scores.

### 2.11 Sample size calculation

The sample size was determined based on the primary outcome. The team conservatively anticipated a medium effect size for the group difference (Cohen's *f* ≈ 0.175, which corresponds roughly to d ∼0.35–0.40 in pairwise terms) given prior results based on the prior study on healthy individuals (11). Power analysis (*α* = 0.05 two-tailed, 1 − *β* = 0.80) for a one-way analysis of covariance (ANCOVA) with 4 groups and baseline as covariate, assuming the covariate explains ∼20% of variance, indicated needing about N ≈ 105 participants per group to detect *f* = 0.175. Allowing for up to ∼35% dropout, due to the fully online nature and 8-week duration, we decided to increase the target enrollment to ensure at least 105 completers per arm. Including the two extra groups for the conditioning test, the total planned sample size is *N* = 630 (105 × 6). This large sample will provide adequate power not only for primary comparison but also to explore secondary endpoints. Interim analyses are not planned; all data will be analyzed after study completion. The qualitative data from open-ended questions will be analyzed using deductive-inductive qualitative content analysis to identify patterns and themes in participants' experiences. We will integrate quantitative and qualitative findings in interpretation to understand the intervention's impact under consideration of multiple perspectives.

### 2.12 Biosignal processing

A total of 15 min of ECG (130 Hz) and photoplethysmographic (PPG; 28 Hz) data will be collected from all participants of the sub-study during the resting conditions at T0 and T1. The ECG data will be recorded using a Polar H10 chest-strap, and the PPG data will be recorded using a Polar Verity Sense optical pulse sensor. The participants will remain in a seated position during the recording. The time series estimating functions of the autonomic regulation will be semi-automatically extracted from the raw data using Kubios HRV Scientific Software for the ECG and an in-house software (programming environment Matlab R2025a) for the PPG, derived as follows:

- time series of heart rate, consisting of successive beat-to-beat intervals [BBI, (ms)],
- time series containing maximum PPG peak amplitudes per heartbeat [PPGMAX, (a.u.)], and
- time series containing minimum PPG peak amplitudes per heartbeat [PPGMIN, (a.u.)].

Afterwards each 15-min time series will be filtered by applying an adaptive variance estimation algorithm to remove and interpolate ectopic beats and artefacts to ensure normal-to-normal time series (NN) (21). Afterwards indices describing autonomic regulation will be calculated in the time domain (TD), in the geometric domain [Baevsky's stress index (22)], the frequency domain (FD) (the power spectra of equidistant linear interpolated NN interval time series will be obtained by applying the Fast Fourier Transformation), and in the nonlinear domain (NLD) (Table 1).

**Table 1:** Definition of clinically relevant HRV indices and their assignment to the autonomic nervous system.

Domain	Index	Unit	Definition and explanation	Activity as part of the autonomic nervous system
Time domain	meanNN	ms	Mean value of all NN intervals	—
	SDNN	ms	Standard deviation of NN intervals in the measurement time range	Sympathetic nervous system and parasympathetic nervous system
	SDANN	ms	Standard deviation of the average of NN intervals in 5-min segments	Sympathetic nervous system and parasympathetic nervous system
	RMSSD	ms	“Root Mean Square of successive differences” (Square root of the mean value of the sum of all squared differences between adjacent NN intervals)	Parasympathetic nervous system
	NN50	beats	Number of pairs of adjacent NN intervals that differ by more than 50 ms	Parasympathetic nervous system
	pNN50	%	Percentage of consecutive NN intervals that differ by more than 50 ms	Parasympathetic nervous system
Geometric	SI		Stress Index, square root of Baevsky stress index, reflects cardiovascular stress	Sympathetic nervous system
Frequency domain	TP	ms^2^	Total Power: Total power or total spectrum; corresponds to energy density in the spectrum from 0.00001 to 0.4 Hz (VLF, LF and HF)	No clear assignment
	VLF	ms^2^	“Very low frequency power” (Power density spectrum in the frequency range of 0.003–0.04 Hz)	Parasympathetic nervous system
	LF	ms^2^	“Low frequency power” (Power density spectrum in the frequency range of 0.04–0.15 Hz); LFn [n.u.]	Sympathetic and parasympathetic nervous systems, with the parasympathetic nervous system predominating
	HF	ms^2^	“High frequency power” (Power density spectrum in the frequency range of 0.15–0.40 Hz); HFn [n.u.]	Parasympathetic nervous system
	LF/HF	—	Ratio of LF and HF	Sympathetic-parasympathetic balance
Nonlinear dynamics Poincaré-Plot	SD1	ms	Standard deviation of the point distances to the transverse diameter	Parasympathetic nervous system
	SD2	ms	Standard deviation of the point distances to the longitudinal diameter	Sympathetic nervous system and parasympathetic nervous system

In NLD most prominent measures will be applied describing important features such as nonlinearity, time irreversibility, fractality, and long-range correlations of physiological dynamics. As one example described in more detail, Symbolic dynamics (SD). The idea of SD is based on simplifying the non-linear dynamics (the way things change over time) that are part of time series (23). Consequently, the NN will be transformed into a symbol sequence of four symbols with a given alphabet *A* = {0, 1, 2, 3}. The creation of words from this new alphabet (consisting of a symbol string) will be achieved through the utilization of three successive symbols, thereby enabling 64 distinct word-type combinations (bins) (000, 001,…,333). The following SD indices from the probability distribution of each word-type within the symbol sequence will be estimated:

- wpsum02: Relative portion (sum/total) of words consisting only of the symbols “0” and “2”, a measure for reduced variability [a.u.], and
- wpsum13: Relative portion (sum/total) of words consisting only of the symbols “1” and “3”, a measure for increased variability [a.u.].

Further NLD HRV which will be applied are Poincaré plot analysis (PPA) (24), Approximate (ApEn) and Sample entropy (SampEn) (25), Multiscale entropy (MSE) (26) and Detrended fluctuation analysis (DFA) (27) (Table 1). Although TD and FDmethods facilitate the quantification of HRV on disparate time scales, NLD methods furnish supplementary information with regard to the dynamics and structure of beat-to-beat time series. Notwithstanding, the disadvantage associated with these methodologies partly remains the complexity involved in the physiological interpretation of the results (23, 28).

### 2.13 Data analysis

The primary outcome (MDBF calmness subscale change from T0 to T1) will be analyzed using an ANCOVA model. The model will include the study group (4 arms for primary analysis: Groups 1–4) as the independent variable and the baseline MDBF score as a covariate to control for any initial differences and improve statistical power. This aligns with the principle of analyzing covariance to account for baseline variance. The primary contrast of interest is whether either of the two combination groups (1: Hypnosis + Bergamot, 2: Hypnosis + Lavender) differ significantly from the control groups (3: Hypnosis-only, 4: Minimal intervention) in adjusted mean relaxation at T1. We anticipate a significant overall group effect; planned pairwise comparisons with appropriate correction for multiple comparisons, if needed, will examine specific differences, especially 1 vs. 3, 2 vs. 3, and combined 1&2 vs. combined 3&4. The primary hypothesis will be supported if Groups 1 and 2 show statistically greater increases in relaxation than Group 3 and Group 4 (*p* < .05, two-tailed).

For Phase 2 conditioning effects, a similar ANCOVA or mixed-model approach will be used at T2. The analysis will focus on the four groups that underwent Phase 2 aroma testing (Groups 1, 2 with match vs. Groups 5, 6 with mismatch). We will compare relaxation outcomes at T2 among these, using T1 values as covariates. A significant difference (e.g., match > mismatch) on the MDBF relaxation change from T1 to T2 or on the Phase 2 VAS responses would support H2.1. We may also use repeated measurements ANOVA or multilevel modeling incorporating all three time points (T0, T1, T2) to examine group × time interactions across the full study duration. The VAS data from each session will be analyzed using linear mixed-effects models, which can handle multiple observations per participant. These models will test differences in immediate relaxation gain per session by group and can also model time trends (session number as a predictor) to see if there is a significant upward trend in pre-session relaxation over the 6 weeks for each group (learning effect).

All secondary continuous outcomes (anxiety, stress, sleep, well-being, worry scores) will similarly be analyzed via ANCOVA at T1 and T2 controlling for baseline, comparing the four main groups. We expect trends favoring the combined intervention, but these will be interpreted with caution and adjusted for multiple comparisons since multiple scales are involved. Moderator analyses (H2.3) will be exploratory by including interaction terms in the ANCOVA or regression models or conduct subgroup analyses splitting the sample by median age. Given the sample size (∼105 per arm planned), the trial is powered primarily for the main effect on the primary outcome; subgroup effects would have to be large to be detectable, so these findings will be descriptive.

### 2.14 Qualitative analysis

A pragmatic qualitative content analysis with a combined deductive-inductive approach (29) is conducted using the software MAXQDA 2026 and oriented to the procedure outlined by Rädiger and Kuckartz (30): Responses to the open-ended questionnaire items are imported into the software and initially reviewed to gain an overview of the material and to familiarize the researchers with the data. Deductive categories are developed based on the predefined themes of the open-ended questions, while allowing for the inductive emergence of additional themes from the data. The coding framework is iteratively refined during the analysis and discussed within the research team to enhance intersubjective comprehensibility as a key quality criterion of qualitative research (31). The aim of the qualitative analysis is to synthesize the core themes emerging from participants’ experiences with the intervention.

## 3 Discussion

This study is a comprehensive trial examining a novel multimodal intervention that integrates digital self-hypnosis with essential oil aromatherapy for individuals suffering from persistent worries and stress at a subclinical level. By leveraging the anxiolytic properties of lavender and bergamot essential oils in tandem with the deep relaxation of hypnosis, the study aims to enhance stress reduction outcomes and potentially condition a natural relaxation trigger, the essential oil, that participants can use beyond the intervention. The significance of this research lies in its preventive focus, because helping those with mild anxiety manage their symptoms effectively and possibly avert progression to a clinical disorder (1). If successful, the intervention could be a low-cost, easy to use tool, the essential oils are inexpensive, and the hypnosis can be delivered via an app or website, empowering individuals to self-regulate anxiety and stress in their daily lives.

Furthermore, the study will contribute to the scientific understanding of mind-body interactions, confirming whether the addition of a sensory cue can reinforce hypnotic relaxation and create lasting conditioned effects. Previous evidence shows that pleasant odors can directly influence mood and physiology (3–5, 32) and pairing them with therapy techniques may harness the brain's associative learning to prolong benefits. The outcome of this study will inform not only clinical practice in terms of combining aromatherapy with psychological interventions, but also theoretical models of how contextual cues such as essential oil can become safety signals or relaxation triggers through conditioning. A positive finding would open avenues for personalized aromatherapy in mental health, such as patients could choose an essential oil, they find comforting to integrate into their therapy, thus creating a portable cue for calm that they carry into real-world situations. The anticipated conditioning effects of lavender and bergamot essential oils are assumed to arise from their olfactory properties and the associated scent stimulus that elicit the conditioned response. Potential stress-reducing and anxiolytic pharmacological effects of the oils' bioactive constituents are not accounted for and may, however, have contributed to the observed effects that are attributed to conditioning.

An additional objective index of stress physiology would be salivary cortisol (e.g., diurnal profiles or the cortisol awakening response) (33). While such endocrine assessments were beyond the scope of the present trial, future trials building on this foundation will integrate multimodal biomarkers to more comprehensively characterize psychophysiological mechanisms and intervention effects.

In summary, this study addresses an important gap for those with subthreshold anxiety, testing an innovative approach to bolster their coping resources. By rigorously evaluating subjective, psychometric, and objective physiological outcomes, this study will clarify the efficacy of combining essential oil inhalation and self-hypnosis and examine the durability of its effects via conditioned associations. As anxiety and stress continue to be widespread issues, especially in modern fast-paced life, accessible interventions that promote relaxation and resilience are in high demand (34). This trial's findings will shed light on whether an “essential oil-conditioned hypnosis” approach can be one such effective strategy to help individuals cultivate lasting calm and improve their overall well-being. The integration of quantitative results with qualitative insights will also reveal how participants experience the intervention in daily life, guiding future refinements. Ultimately, if the hypotheses are confirmed, the combination of guided self-hypnosis and essential oils could become a practical, evidence-based method for distress reduction and mental health promotion in subclinical populations, with potential to be scaled broadly through digital health platforms.

## 4 Ethics and dissemination

The study has been approved by the responsible ethics committee, and all participants provide informed consent prior to participation. The intervention is designed as a low-intensity, non-invasive digital self-help program targeting individuals with subsyndromal anxiety and stress. Guided self-hypnosis exercises are delivered in a standardized, supportive format and do not involve exposure to traumatic material or symptom provocation. Participants are informed that the intervention is not intended to replace clinical treatment and are advised to seek professional help if symptoms worsen or if clinically relevant anxiety emerges during the study. Clear information on withdrawal rights and contact options for study staff is provided throughout participation.

Potential risks associated with self-hypnosis and essential oil inhalation are considered minimal. Participants are screened for contraindications to hypnosis and essential oil use, and safety instructions regarding essential oil inhalation are provided. Adverse events will be monitored and documented throughout the study period. For the optional physiological assessments conducted in Berlin, standard safety procedures for ECG and pulse recordings are followed.

To ensure participant safety, individuals whose screening results fall close to or exceed predefined cut-off values for generalized anxiety will undergo an additional eligibility assessment prior to study inclusion. These participants will be invited to a brief virtual screening meeting with a psychologist or medical staff of the study team to clarify symptom severity, current treatment status, and potential risk factors. Study participation will only commence if it is determined that inclusion is safe and appropriate within the scope of a low-intensity, self-guided intervention. Participants for whom clinically relevant anxiety or safety concerns are identified during this secondary screening will not be enrolled and will be advised to seek appropriate professional support.

Study results will be disseminated through peer-reviewed scientific publications and conference presentations, regardless of outcome. The trial will be reported in accordance with CONSORT guidelines for randomized trials (35). In addition, aggregated and anonymized findings may be shared with participants and the broader public through accessible summaries. This dissemination strategy aims to promote transparency and support the responsible development of scalable digital interventions for stress and anxiety prevention.
